# Finlay–Wilkinson random regression for yield and yield stability prediction in cereals

**DOI:** 10.1002/tpg2.70269

**Published:** 2026-09-09

**Authors:** Pablo Sandro, Justin Blancon, Jeffrey Neyhart, Julie Dawson, Lucia Gutiérrez

**Affiliations:** ^1^ Department of Plant and Agroecosystem Sciences University of Wisconsin Madison Wisconsin USA; ^2^ UMR GDEC, INRAE, Université Clermont Auvergne Clermont‐Ferrand France; ^3^ USDA‐ARS, Genetic Improvement for Fruits & Vegetables Laboratory Chatsworth New Jersey USA; ^4^ Department of Plant Breeding Swedish University of Agricultural Sciences Alnarp Sweden

## Abstract

Year‐to‐year climate variability poses a challenge for agriculture by increasing crop yield variability; therefore, there is a need to identify genotypes that can withstand these fluctuations. With the right selection criteria, genotypes with yield stability across variable environmental conditions can be selected. Methods such as Finlay–Wilkinson random regression (FWRR) may allow us to use sparse datasets—common in plant breeding pipelines—and incorporate genomic data to leverage phenotypic information from related genotypes to predict yield stability. Our objective was to examine how the number of environments and the variance among those environments affect stability predictions. We also integrate FWRR as a genomic prediction tool for characterizing yield stability, comparing it to the traditional genomic prediction models as a reference. We used three datasets: one highly unbalanced dataset for oats (*Avena sativa* L.) and two completely balanced datasets with different numbers of environments for barley (*Hordeum vulgare* L.) and wheat (*Triticum aestivum* L.). We fit standard Finlay–Wilkinson (FW) and FWRR models to estimate grain yield and stability under various scenarios. We found that the estimated stability values obtained were similar using balanced datasets for FW or FWRR. FWRR also achieved moderate predictive ability for stability using unbalanced datasets under 10‐fold cross‐validation (CV1) with new genotypes. In terms of environmental representation, selecting the right set of environments for inclusion in the model was more important than adding more environments. Our results suggest the possibility of using FWRR to select stable genotypes earlier in line development, as well as to design resource‐efficient stability‐testing schemes.

## Plain Language Summary

Farmers face challenges due to cultivar responses to weather variability. Some cultivars handle these changes better than others, so it is important to identify cultivars that produce stable and high yields across different conditions. Many methods exist to measure stability, but they often require complete datasets from many locations, and breeding programs rarely have this kind of perfect information. In this study, we tested whether information from related cultivars can help estimate a cultivar's stability, even when the datasets are incomplete. We examined the number of environments required for accurate stability estimates; applied the approach to oats, barley, and wheat; and demonstrated that both complete and incomplete datasets enable reliable stability prediction. We also learned that about 20 environments are needed, but choosing the right locations matters as much as adding more locations. These results can help breeders select stable, high‐performing cultivars.

**Abbreviations**
BLUE: best linear unbiased estimator
BLUP: best linear unbiased predictor
CV1: 10‐fold cross‐validation
FW: Finlay–Wilkinson regression
FWRR: Finlay–Wilkinson random regression
gBLUP: genomic best linear unbiased predictor
GEI: genotype‐by‐environment interaction
MET: multi‐environment trials
TPE: target population of environments

## 1 INTRODUCTION

The impact of global climate change on climate variability alters the frequency and duration of biotic (i.e., weeds, pests) and abiotic (i.e., drought, floods, heat) stresses, challenging agricultural production worldwide (Alotaibi, 2023; Intergovernmental Panel on Climate Change, 2023). Therefore, plant breeding programs need to constantly update their goals to develop varieties adapted to future climatic conditions (Paniza, 2024). Climate change is expected to reshape plant breeding and selection objectives in unknown and unprecedented ways (Xiong et al., 2022). In addition, Macholdt and Honermeier (2017) found that farmers and scientists expect phenotypic stability to become more relevant in the coming years due to climate change. Given the inherent difficulty of predicting environmental conditions, breeding programs need to adapt and develop strategies to deal with climatic variability (Alotaibi, 2023). Dynamic stability, also termed type I stability in the plant breeding literature, is the consistent performance of a genotype relative to other varieties tested in the same set of environments (Annicchiarico, 2002; Becker & Léon, 1988). To select for stable genotypes, plant breeders need selection tools that integrate genotype‐by‐environment interactions (GEIs) and stability into breeding decisions (Alseekh et al., 2025; Sandro et al., 2022). These tools will facilitate the development of stable cultivars that can withstand climatic stresses (Picasso et al., 2019).

An environment, in the context of multi‐environment trials (METs), is defined as a specific combination of location, year, and management or treatment conditions (Bernardo, 2014). This concept encapsulates the combined effects of biotic and abiotic factors that influence the phenotype, which results from the expression of the genotype, the environment, and their interaction. Genotypes under evaluation in a breeding program are tested at multiple locations over several years to capture genotype‐by‐environment interactions (Piepho & Möhring, 2006; Yan, 2015). The locations selected for a METs represent a repeatable sample of the possible locations within the target population of environments (TPEs) for a breeding program (Yan et al., 2011). On the other hand, the effects of year and location‐by‐year interactions are non‐repeatable, inherently difficult to predict, and account for a significant portion of the overall variance in phenotypic performance (Yan et al., 2011). The TPE represents the repeatable and unrepeatable effects that explain GEIs in the region where breeders intend cultivars to be grown. Testing locations are ideally chosen based on the quality oif the data that they provide over time (Neyhart et al., 2022) but are also constrained on the availability of testing infrastructure (Van Eeuwijk et al., 2016). Although breeders typically recommend using locations that represent the average range of variation within the TPE, environments outside the TPE can also be useful when they expose genotypes to specific or controllable abiotic stresses that are infrequent but important to assess (Bernardo, 2014). On the other hand, anomalous environments are typically detrimental to the use of MET data for phenotypic selection or genomic prediction (Heslot et al., 2015). Nevertheless, environmental conditions that were once outliers are becoming more common, and breeding programs must develop selection tools that account for this and select for stable genotypes capable of withstanding this variability.

Breeding programs are in a perpetual struggle to use their resources most efficiently to obtain the best phenotypic information for as many genotypes as possible in the shortest time possible (González‐Barrios et al., 2019; Hoefler et al., 2020; Lado et al., 2018; Rebollo et al., 2023). At most evaluation stages, breeders face a trade‑off between the number of locations assigned to each genotype and the number of genotypes evaluated (Gauch & Zobel, 1996). As a consequence, genotypes are typically evaluated in a subset of the total environments available for testing (Gonzalez‐Barrios et al., 2019; Piepho & Möhring, 2006; Smith et al., 2001). This results in hierarchical trials in which genotypes that do not meet performance targets are discarded in subsequent years of testing, generating unbalanced datasets over time (Piepho & Möhring, 2006). The extent to which environmental factors influence a trait and a GEI determines the level of phenotypic testing required to differentiate genotypes that meet the requirements of the target population of environments (Hoefler et al., 2020). For traits with high heritability and limited environmental influence, breeders require fewer test environments to obtain accurate estimates of genotypic performance. Quantitative traits, such as yield, however, which show a strong degree of environmental influence and GEIs, require the highest level of testing across the TPE with a rigorous experimental design (Bernardo, 2010). In particular, grain yield stability is a challenging trait to estimate due to the number of environments required to achieve acceptable accuracy (Wang et al., 2023), and there is no consensus on how many environments are needed for accurate estimates (Reckling et al., 2021). The number of environments will change depending on the heritability estimates achieved in the trials (Yan et al., 2015). Estimates in the literature for the number of environments needed range from 21 for characterizing GEIs in oats (Yan et al., 2015) to at least 150 for estimating stability in physiological traits in wheat (Wang et al., 2023). In other crops, such as corn (*Zea mays* L.), 50–200 environments are suggested (Piepho, 1998a, 1998b). Consequently, the large number of environments required makes grain yield stability a complex and resource‑intensive trait to phenotype.

The complexity of evaluating grain yield stability poses a particular limitation in self‑pollinating species such as oat (*Avena sativa* L.), barley (*Hordeum vulgare* L.), and wheat (*Triticum aestivum* L.), which require multiple generations of self‑pollination to achieve homozygosity. Even when there are well‑established methods to help accelerate the breeding cycle for oats (González‐Barrios et al., 2021), barley (Gopinathan et al., 2025; Watson et al., 2018), and wheat (Bhatta et al., 2021; Dhakal et al., 2025), breeders must still conduct several additional years of evaluation before reaching METs and obtaining the first stability estimates. In a broader sense, the characterization of grain yield stability for a genotype or agricultural system has extensive prior work reported in the literature, including many indices to estimate stability (Pour‐Aboughadareh et al., 2022). However, researchers have not reached a consensus on which estimator to use (Reckling et al., 2021). Therefore, a common practice is to calculate several indices to capture different aspects of stability (i.e., Bocci et al., 2020; Mohammadi & Amri, 2013; Mühleisen et al., 2014). Most stability indices require complete, balanced data in which all genotypes are evaluated across all environments (Bacsi & Hollósy, 2019; Francis & Kannenberg, 1978; Gauch, 1992; Lin & Binns, 1988; Wricke, 1962; Yan & Kang, 2002). Other indices, such as β from the Finlay–Wilkinson (FW) regression (Finlay & Wilkinson, 1963), offer greater flexibility and allow the use of unbalanced genotype data to calculate stability (Pour‐Aboughadareh et al., 2022; Turbet Delof et al., 2024). When the number of genotypes in an MET is large, another option is to model genotypes and environments as random factors using Finlay–Wilkinson random regression (FWRR), which can account for the lack of independence between genotypes and environments (Piepho & Blancon, 2023). Being able to predict individual genotype slope accurately (${\tilde{b}}_{i}$) may allow us to select for grain yield stability, which is an important trait for adaptation to future climatic uncertainty (Alseekh et al., 2025). Furthermore, researchers can use FWRR for early characterization in self‑pollinated crops by leveraging historical and sparse multi‑environment trial data, as well as data from other contexts, such as on‑farm trials, through genetic relationships among genotypes and genetic correlations among environments.

More recently, the availability of genomic information at a low cost allows for further optimization of resource allocation across locations without losing data quality (Endelman et al., 2014; Hoefler et al., 2020). Genomic information also enables the use of historical MET data to understand GEIs (González‐Barrios et al., 2019; Sandro et al., 2024). Genomic prediction also offers new strategies to understand stability (Heslot et al., 2015). With the fast development and availability of extensive genomic information (Bekele et al., 2024), several genomics strategies have been proposed over time to include multi‐environment data in genomic contexts (Li & Gutiérrez, 2023), such as using prediction models with unbalanced data to calculate stability indicators (Carvalho et al., 2024) using linear regressions (Neyhart et al., 2025), Bayesian joint‐regression, or combining genomic data with FWRR (Ly et al., 2018; Piepho & Blancon, 2023).

Our goal in this study was to compare how FWRR and FW are affected by the number of environments used to fit the model and by the variance among the environments' means, and to examine how these factors affect the results observed in each model. We also aimed to test a model integrating FWRR with genomic data to determine if we could accurately predict yield and yield stability using unbalanced training datasets. We compared its performance with standard FW regression models, the genomic best linear unbiased predictor (gBLUP) basic genomic model, and the factor analytic genomic prediction model to model GEIs.

## 2 MATERIALS AND METHODS

### 2.1 Germplasm and experiments

We used datasets with best linear unbiased estimator (BLUE) at the environment level for grain yield for three crops: oats (Figure S1), initially reported by Sandro et al. (2024); barley (Figure S1), initially reported by Neyhart et al. (2019); and wheat (Figure S1), initially reported by Rincent et al. (2019).

For oats, a highly unbalanced dataset was generated between 1997 and 2021 across 59 locations in seven Midwest states in the United States. The trials are part of the United States Department of Agriculture‐coordinated collaborative nursery and include public breeding program data across the United States and Canada, where oats have been traditionally grown. We used data from the Midwestern United States only; all data are available in the T3/Oat database (https://oat.triticeaetoolbox.org). For the oat training data, 17 environments (location–year combinations) with heritability below 0.2 were considered poor‐quality environments and were removed from the training data (Figure S2). We genotyped 581 oat genotypes using the 3k multispecies chip developed by Fiedler et al. (2024), yielding 2989 markers. All genotypes had more than 80% of marker data. For the genetic markers, we removed 175 markers with a missing‐data proportion greater than 30%, 348 markers with a minimum allele frequency lower than 0.01, and 11 markers with an observed heterozygosity greater than 0.95. The final marker matrix had 3.41% of missing information, 581 genotypes, and 2459 SNPs markers; we did not impute these markers. We then split it into two phenotypic datasets for oats, the original “unbalanced” dataset, which includes all genotypes evaluated in at least three environments, and the “balanced” dataset composed of a subset of individuals that where tested in at least 20 environments; this dataset is not truly balanced and only called in this way as a convention to differentiate them easily in the text.

The barley dataset is a subset of the dataset from Neyhart et al. (2019) in which we removed five environments with only 50 genotypes to achieve a balanced dataset. The final dataset was generated between 2016 and 2017 at 17 locations across the Northern United States and Southern Canada, representing areas where barley has traditionally been grown.

The wheat dataset is balanced and was generated in 2012, 2013, and 2014 across 14 locations, testing water and nitrogen use efficiency across France (Rincent et al., 2019). The realized additive relationship matrix was calculated using genetic markers available for each dataset (Table 1 and Figure S2) following Van Raden (2008).

**TABLE 1:** Description of oat, barley, and wheat datasets, including number of genotypes, number of environments (combination of location and year), number of environments for each genotype, mean heritability for grain yield (range in parentheses), and number of markers.

Dataset	Number of genotypes	Number of environments	Number of environments per genotype	Heritability of grain yield	Number of SNP markers
Oat	581	673	3–480	0.46 (0.20–0.94)	2459
Barley	223	30	28–30	0.68 (0.18–0.86)	6361
Wheat	210	16	16	0.86 (0.44–0.95)	34,464

Abbreviation: SNP, single nucleotide polymorphism.

For additional details about each dataset, see Neyhart et al. (2019), Rincent et al. (2019), and Sandro et al. (2024). In all datasets, the trials used had medium‐to‐high heritability: oat heritability was estimated for each environment using the oat database that contained plot level data, with narrow‐sense entry mean heritability modeled using the additive relationship matrix and the error variance matrix from stage 1. The barley heritability was reported by Neyhart et al. (2019), and the wheat heritability was reported by Rincent et al. (2019, Table 1, and Figure S2).

### 2.2 Environmental means

The mean yield performance for each environment was obtained as the BLUE using the following model:

(1) $$y_{ij}=\mu +G_{i}+E_{j}+GE_{ij}+\varepsilon_{ij},$$

where$\;y_{ij}$ is the BLUE for grain yield for the *i*th genotype in the *j*th environment, $\;G_{i}$ is the effect of the *i*th genotype, $E_{j}$ is the effect of the *j*th environment (i.e., treatment–year–location combination), $GE_{ij}$ is the effect of the interaction between the *i*th genotype and the *j*th environment, and $\varepsilon_{ij}$ the residual error associated with the *i*th genotype in the *j*th environment with $\varepsilon_{ij}$∼ N(0, $\sigma_{\varepsilon }^{2}$) where $\sigma_{\varepsilon }^{2}$ is the residual variance. The inverse of the error variance $\sigma_{\varepsilon }^{2}$ from the first stage BLUEs analysis was used as initial values to model the error variance in the second stage (Butler et al., 2020; Damesa et al., 2017; Smith et al., 2001) in Equations ((1), (2), (3), (4)). Environmental means were estimated whenever the genotype composition changed within a given environment (cross‐validation and simulated scenarios) to maintain a representative genetic correlation between environments and their means.

### 2.3 FW regression

We fitted an FW regression of genotypic performance over environmental performance, with the environmental performance estimated as the mean grain yield for that environment, calculated as described above, with parameters for genotype and environment fitted as fixed effects using the following model:

(2) $$y_{ij}=\alpha_{i}+\beta_{i}w_{j}+\varepsilon_{ij},$$

where $y_{ij}$ is the grain yield BLUEs for the *i*th genotype in the *j*th environment, $\alpha_{i}$ is the regression intercept for the *i*th genotype, $\beta_{i}$ is the regression slope for the *i*th genotype, $w_{j}$ is the environmental performance estimated as described in the previous section, and $\varepsilon_{ij}$ is the residual error associated with the *i*th genotype in the *j*th environment, with $\varepsilon_{ij}$∼ N(0, $\sigma_{\varepsilon }^{2}$). The model and all subsequent models were fitted using the ASReml‐R package (Butler et al., 2020) of the R statistical software (R Development Core Team, 2017)

### 2.4 Finlay–Wilkinson random regression

We estimated FWRR to leverage information from relatives tested in other environments using the following linear mixed model:

(3) $$y_{ij}=g_{i}+b_{i}w_{j}+\varepsilon_{ij},$$

where $y_{ij}$ is the grain yield BLUEs for the *i*th genotype in the *j*th environment, with $g_{i}\;\mathrm{and}\;b_{i}$ as random effects, where $g_{i}$ is the random regression intercept for the *i*th genotype; $b_{i}$ is the random regression slope to the *i*th genotype; $w_{j}$ is the environmental performance estimated as described in the previous section; and $\varepsilon_{ij}$ is the residual error associated with the *i*th genotype in the *j*th environment, with $g_{i}∼\;\mathrm{MVN}\left(\mu_{g},\sigma_{g}^{2}K\right)$, $b_{i}∼\;\mathrm{MVN}\left(\mu_{b},\sigma_{b}^{2}K\right)$, where K is the realized additive relationship matrix calculated using VanRaden (2008), and $\varepsilon_{ij}$∼ N(0, $\sigma_{\varepsilon }^{2}$). The covariance between $g_{i}$ and $b_{i}$, $\;\sigma_{g_{i}b_{i}}^{2}$ was modeled as $\;\sigma_{g_{i}b_{i}}^{2}K$ (Piepho & Blancon, 2023).

### 2.5 gBLUP model

To compare the FWRR and FW models to a standard genomic prediction method without including environmental factors, we fitted a gBLUP model (Bernardo, 1994) using the following linear mixed model:

(4) $$y_{ij}=\mu +g_{i}+\varepsilon_{ij},$$

where $y_{ij}$ are the genotypic BLUEs for grain yield of the *i*th genotype in the *j*th environment (i.e., treatment–location–year combination); μ is the overall mean or intercept; $g_{i}$ is the effect of the *i*th genotype $g_{i}∼\;\mathrm{MVN}\left(0,\sigma^{2}K\right)$, where K is the realized additive relationship estimated matrix; and $\varepsilon_{ij}$ is the residual error with $\varepsilon_{ij}\;∼$ N(0, $\sigma_{\varepsilon }^{2}$). The gBLUP is equivalent to the $g_{i}$ FWRR model component if we equal $w_{j}$ to zero.

### 2.6 Factor analytic model

To compare the FWRR and FW models to genomic prediction method that models GEIs, we fitted a factor analytic model using the following linear mixed model:

(5) $$y_{ij}=\mu +g_{i}+E_{j}+ge_{ij}+\varepsilon_{ij},$$

where $y_{ij}$ are the genotypic BLUEs for grain yield of the *i*th genotype in the *j*th environment (i.e., treatment–location–year combination); μ is the overall mean or intercept; $g_{i}$ is the effect of the *i*th genotype $g_{i}∼\;\mathrm{MVN}\left(0,\sigma^{2}K\right)$, where K is the realized additive relationship estimated matrix; $E_{j}$ is the effect of the *j*th environment (mega environment effect in the case of oats); $ge_{ij}$ is the *i*th genotype by the jth environment interaction with $ge_{ij}∼MVN\left(0,\Sigma \right)$; and $\varepsilon_{ij}$ is the residual error with $\varepsilon_{ij}\;∼$ N(0, $\sigma_{\varepsilon }^{2}$). Here, Σ is the variance–covariance matrix of the GEI, with $\Sigma_{\left(\left[\mathrm{gxe}]x[\mathrm{gxe}\right]\right)}=\Sigma_{G}\;⊗\Sigma_{E}$, where $\Sigma_{G(\mathrm{gxg})}$ is **K**, the realized additive relationship matrix and $\Sigma_{E(\mathrm{exe})}$ is the genotypic variance–covariance matrix among environments modeled as a factor‐analytic of order 1 (FA1).

### 2.7 Effect of the number of environments on regression slopes (*β* and *b*)

#### 2.7.1 Number of environments in oats

**FIGURE 1:**
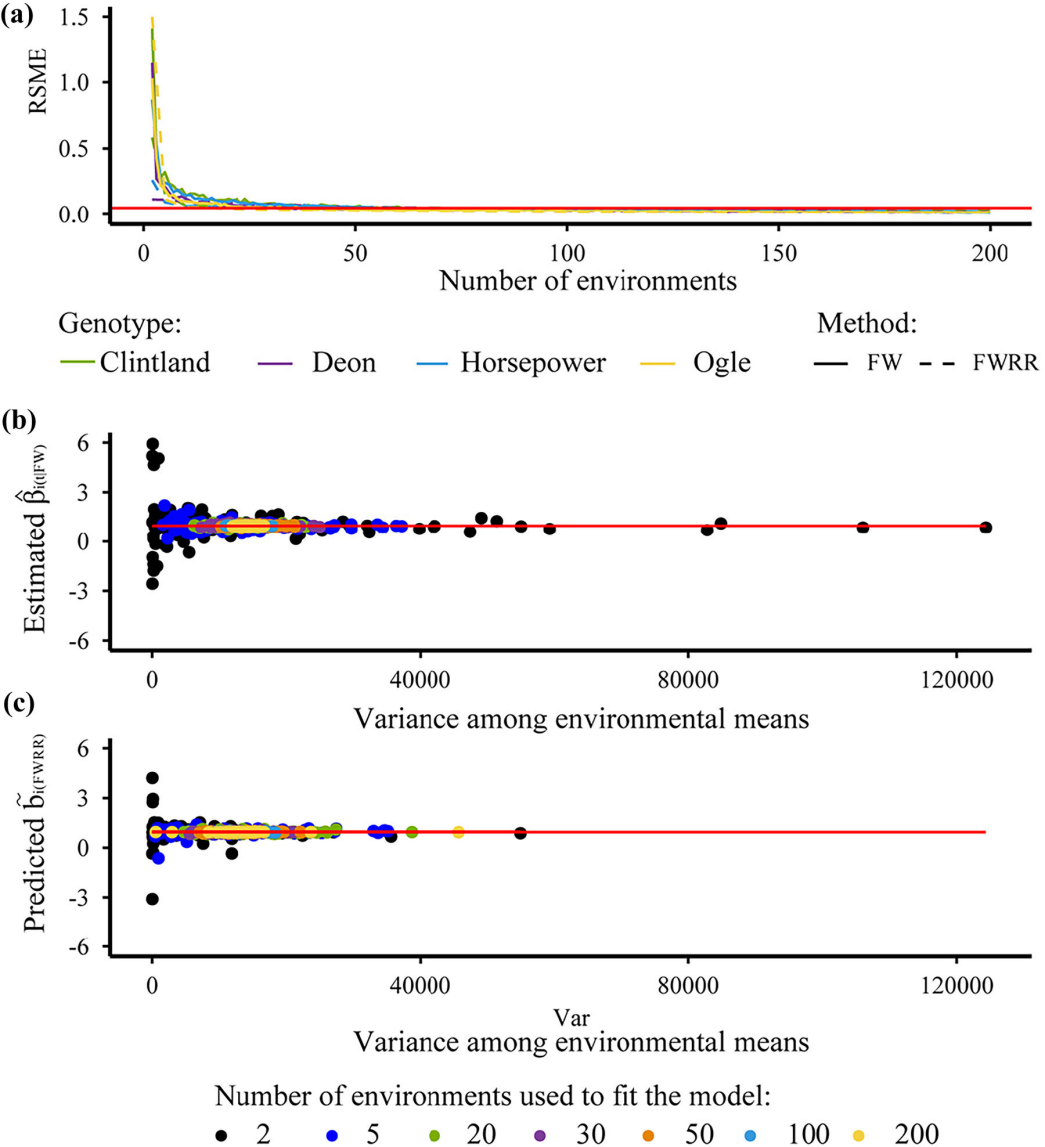
Effect of the number of environments and the variance among the environmental means on stability estimation and prediction in oats. (a) Root mean squared error (RMSE) for stability for the training dataset, comparing the Finlay–Wilkinson (FW) slope (${\hat{\beta }}_{i(t|\mathrm{FW})}$, solid line) and the Finlay–Wilkinson random regression (FWRR) slope (${\tilde{b}}_{i(\mathrm{FWRR})}$, dotted line) as a function of the number of environments in which the genotype is present (100 iterations for each environment number) in the oat data subset. Red line indicates a reference RMSE of 0.05. (b) Estimation of stability (${\hat{\beta }}_{i(t|\mathrm{FW})}$) for “Ogle” using 2–200 environments (100 iterations per environment number), shown as a function of the variance among environmental means in the oat data subset. The red line represents the ${\hat{\beta }}_{i(\mathrm{FW})}$ estimated using the full dataset (${\hat{\beta }}_{i(\mathrm{FW})}$= 0.95, n = 408). (c) Prediction of stability (${\tilde{b}}_{i(\mathrm{FWRR})}$) for “Ogle” using 2–200 environments (100 iterations per environment number), shown as a function of the variance among environmental means in the oat data subset. The red line represents the ${\hat{\beta }}_{i(\mathrm{FW})}$ estimated using the full dataset (${\hat{\beta }}_{i(\mathrm{FW})}$= 0.95, *n* = 408).

To understand the effect of the number of environments on the accuracy of estimates of *β* and *b* in oats, we used two strategies. First, we performed all analyses and simulations on the balanced and unbalanced oat dataset, fitting both models, FW and FWRR. Second, to evaluate the effect of the number of environments, we ran both models, FW and FWRR regressions, and estimated ${\hat{\beta }}_{i(\mathrm{FW})}$ and ${\tilde{b}}_{i(\mathrm{FWRR})}$ using another subset of oat genotypes, in this case, kepping some genotypes that were tested in over 200 environments. For these genotypes, we created training datasets from a random sample of environments from 2 to 200 (i.e., 2, 3, 4, …, 200) across all environments in which the genotypes were present. We performed 100 iterations of this sampling procedure for each number of environments sampled in the progression (i.e., 2–200). For each sample, we estimated the variance among the means of the environments, the range for the means of the environments, and the root mean square error of ${\hat{\beta }}_{i(t|\mathrm{FW})}$ and ${\tilde{b}}_{i(t|\mathrm{FWRR})}$ as a function of the number of environments. For this analysis, we selected four genotypes (“Ogle,” “Horsepower,” “Deon,” “Clintland64”; Figure 1a, Figures S3–S6) because these genotypes were tested in over 200 environments each (just a few genotypes get this level of testing across multiple environments). Therefore, this analysis allows us to have a random sample of environments between 2 and 200 to fit the model and answer the question of how many environments are needed to estimate stability in unbalanced datasets.

#### 2.7.2 Number of environments in barley and wheat

To analyze the effect of the number of environments used to fit the regression on the accuracy of *β* estimation, we subset the available environments to form different training datasets, which we used to fit both FW and FWRR models. We created subsets of 2, 5, and 10 environments in wheat and 2, 5, 10, 15, and 20 environments in barley. We performed 100 iterations for each number of environments sampled. For each iteration, we used only the information from the environments in the subset and masked all information on the remnant environments. For each iteration, we recorded the list of environments, the minimum and maximum environmental means, the environmental mean range, and the variance of environmental means. Based on preliminary results from the barley and wheat testing scenario described above, and to further understand the effect of individual locations on the accuracy of *β* estimation using FW and bi prediction using FWRR, both models were also fitted for all possible pairs of environments within the barley and wheat data.

#### 2.7.3 Estimation of predictive ability of environmental subsets for regression slopes (*β* and *b*)

Predictive ability was calculated as the Pearson correlation cor(${\hat{\beta }}_{i(\mathrm{FW})}$, ${\hat{\beta }}_{i(t|\mathrm{FW})}$) between the FW slope estimated using the full data (${\hat{\beta }}_{i(\mathrm{FW})}$) and the FW slope estimated using the subset of environments (${\hat{\beta }}_{i(t|\mathrm{FW})}$). Predictive ability for FWRR was calculated similarly, with cor(${\hat{\beta }}_{i(\mathrm{FW})}$, ${\tilde{b}}_{i(t|\mathrm{FWRR})}$), where ${\hat{\beta }}_{i(\mathrm{FW})}$ is the slope for the FW model using the full dataset (best known estimator), and ${\tilde{b}}_{i(t|\mathrm{FWRR})}$ is the slope predicted by the FWRR model using data from the subset of environments.

#### 2.7.4 Estimation of predictive ability of environmental subsets for grain yield

To predict grain yield using FWRR, the genotype‐specific predicted slope ${\tilde{b}}_{i(t|\mathrm{FWRR})}$ and intercept ${\tilde{g}}_{i(t|\mathrm{FWRR})}$ were used to solve the regression equation and obtain the predicted grain yield ${\tilde{y}}_{ij(t|\mathrm{FWRR})}$ for each environment in which the genotype was present. Predictive ability (PA) for grain yield was then calculated using only the environments excluded from the training set to evaluate the feasibility of using a reduced set of locations.

To avoid overestimating the correlation between observed and predicted values due to data structure (i.e., Simpson's paradox; Berro et al., 2019), both predicted values (${\tilde{y}}_{ij(t|\mathrm{FWRR})})$ and observed BLUEs ${\hat{y}}_{ij}$ were centered within each environment by subtracting the environmental mean (${\bar{E}}_{j}$). Predictive ability was then calculated as the overall Pearson correlation between environment‐centered observed and predicted values across all environments as $\mathrm{cor}({\hat{y}}_{ij}-{\bar{E}}_{j},{\tilde{y}}_{ij(t|\mathrm{FWRR})}-{\bar{E}}_{j})$, where ${\bar{E}}_{j}$ is the mean of environment j, estimated as described in Equation (1).

The predictive ability for grain yield using FW was calculated following the same procedure: $\mathrm{cor}(\left({\hat{y}}_{ij}-{\bar{E}}_{j}\right)$, $\left({\hat{y}}_{ij(t|\mathrm{FW})}-{\bar{E}}_{j})\right)$. To understand the effect of location on the predictive ability of stability prediction, we grouped the predictive ability by location (Figures S7 and S8) and visualized it using ggplot2 (Wickham, 2016) in R (R Development Core Team, 2017).

#### 2.7.5 FWRR predictive ability using cross‐validation

We performed 10‐fold cross‐validation (CV1; Burgueño et al., 2012) with 100 iterations to evaluate the ability of the FWRR model to predict stability ${\tilde{b}}_{i(\mathrm{FWRR})}$ and grain yield. In the 10‐fold CV1 scheme, genotypes were assigned to one of 10 groups. Predictive ability for the slope of grain yield was then calculated as the correlation (Pearson coefficient) between the best known “true” values (the estimated slope (${\hat{\beta }}_{i(\mathrm{FW})}$) obtained from the full datasets for each genotype) and the predicted slope for those genotypes when they were in the validation group (${\tilde{b}}_{i(\mathrm{cv}1|\mathrm{FWRR})}$), expressed as cor(${\hat{\beta }}_{i(\mathrm{FW})}$, ${\tilde{b}}_{i(\mathrm{cv}1|\mathrm{FWRR})}$). Predictive ability for grain yield was calculated as the correlation between the environment‐centered predicted grain yield and the environment‐centered observed grain yield, cor((${\hat{y}}_{ij}-{\bar{E}}_{j}$),(${\tilde{y}}_{ij(\mathrm{cv}1|\mathrm{FWRR})}-{\bar{E}}_{j}$)).

#### 2.7.6 gBLUP predictive ability using cross‐validation

To compare FWRR yield predictions with the gBLUP model (Bernardo, 1994), we used the same procedures described above to perform a 10‐fold cross‐validation (CV1; Burgueño et al., 2012) with 100 iterations to evaluate predictive ability for grain yield. Predictive ability for grain yield was calculated as the correlation between the predicted grain yield using gBLUP and the observed grain yield in each environment, cor((${\hat{y}}_{ij}-{\bar{E}}_{j}$),(${\tilde{y}}_{i(\mathrm{cv}1|\mathrm{gBLUP})}$)).

#### 2.7.7 Factor analytic predictive ability using cross‐validation

To compare the use of FWRR yield predictions with the FA model (Piepho, 1998a), we used the same procedures described above to perform a 10‐fold cross‐validation (CV1; Burgueño et al., 2012) with 100 iterations to assess predictive ability for grain yield. Predictive ability for grain yield was calculated as the correlation between the predicted grain yield using FA and the observed grain yield in each mega environment in the case of oats, and at the environment level, cor((${\hat{y}}_{ij}-{\bar{E}}_{j}$),(${\tilde{y}}_{i(\mathrm{cv}1|\mathrm{FA})}-{\bar{E}}_{j}$)) for barley and wheat.

#### 2.7.8 Effects of unbalanced data on predictive ability

To observe the effect of unbalanced data on the predictive ability for ${\tilde{b}}_{i(t|\mathrm{FWRR})}$ and ${\tilde{y}}_{ij(t|\mathrm{FWRR})}$, we simulated unbalanced data using a subsample of the barley and wheat datasets. We simulated an unbalanced MET with genotypes present in 25%–80% of the total number of environments, where each environment had at least 33% of the total number of genotypes and 20% variation in the number of genotypes across environments. We used 100 iterations of possible unbalanced data and used a CV1 strategy to evaluate predictive ability. We compared the results to the values from fitting the FWRR with the full balanced dataset and calculated predictive abilities for ${\tilde{b}}_{i(\mathrm{Un}|\mathrm{FWRR})}$, as cor(${\tilde{b}}_{i(\mathrm{FWRR})}$, ${\tilde{b}}_{i(\mathrm{Un}|\mathrm{FWRR})}$) and predicted grain yield ${\tilde{y}}_{ij(\mathrm{Un}|\mathrm{FWRR})}$ as cor((${\hat{y}}_{ij}-{\bar{E}}_{j}$), (${\tilde{y}}_{ij(\mathrm{Un}|\mathrm{FWRR})}-{\bar{E}}_{j}))$.

## 3 RESULTS

### 3.1 Number of environments

**FIGURE 2:**
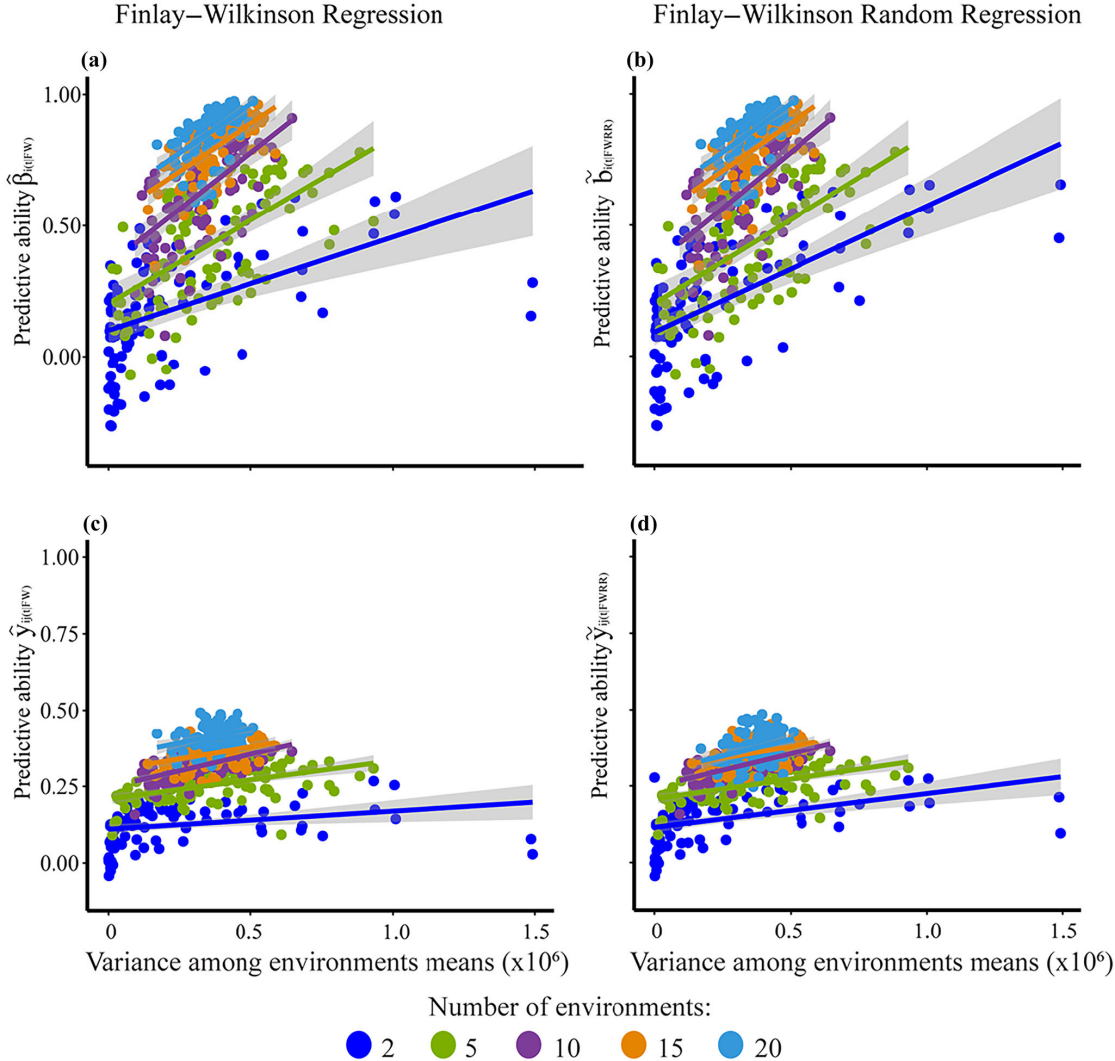
Effect of the number of environments and the variance among environmental means on the predictive ability for stability and grain yield in barley. (a). Finlay–Wilkinson (FW) for stability (${\hat{\beta }}_{i(t|\mathrm{FW})}$). (b) Finlay–Wilkinson random regression (FWRR) for stability (${\tilde{b}}_{i(t|\mathrm{FWRR})}$). (c) FW for grain yield *t* ${\hat{y}}_{i(t|\mathrm{FW})}$). (d) FWRR for grain yield ${\tilde{y}}_{i(t|\mathrm{FWRR})}$.

**FIGURE 3:**
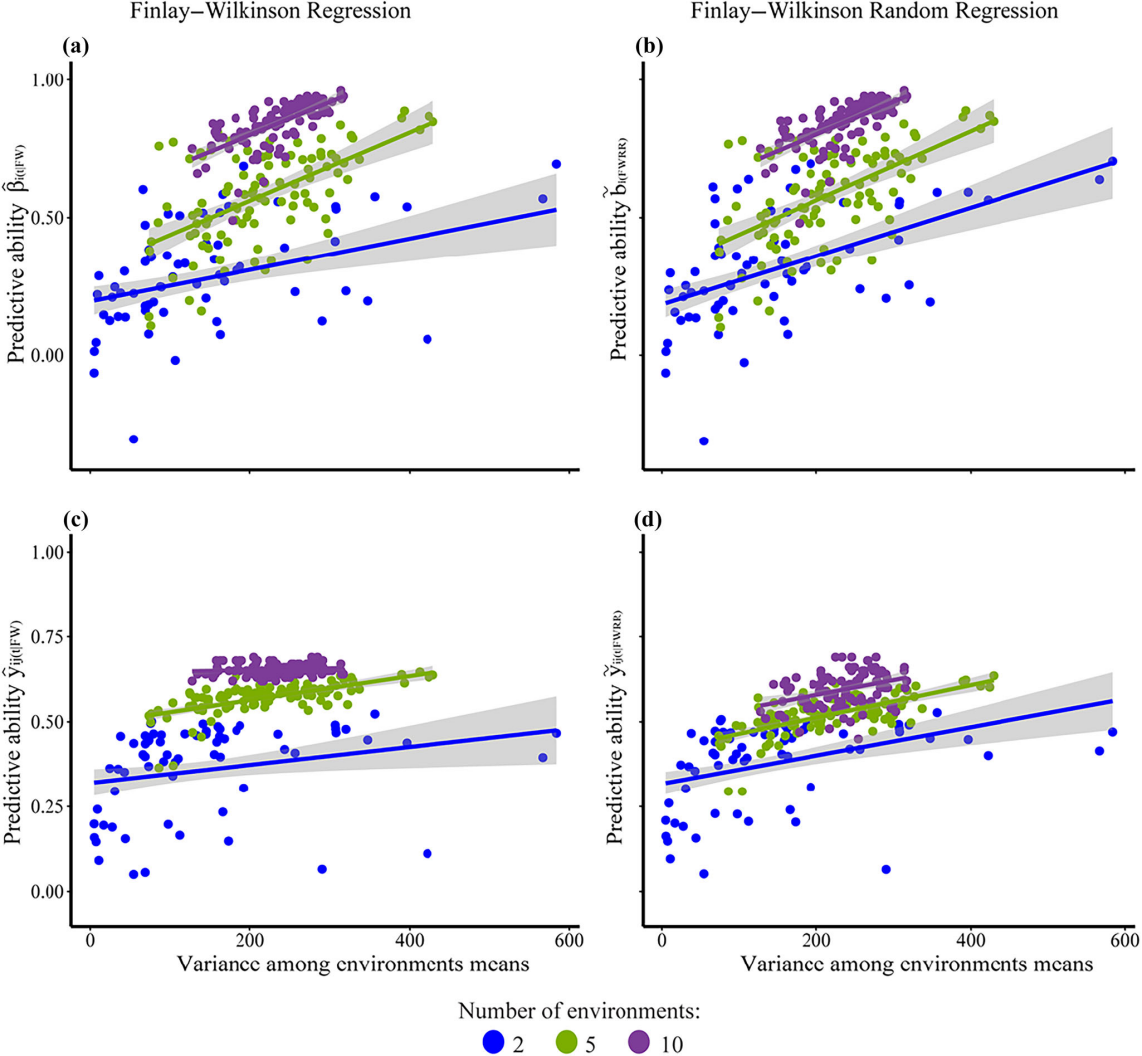
Effect of the number of environments and the variance among environmental means on the predictive ability for stability and grain yield in wheat. (a) Finlay–Wilkinson (FW) for stability (${\hat{\beta }}_{i(t|\mathrm{FW})}$). (b) Finlay–Wilkinson random regression (FWRR) for stability (${\tilde{b}}_{i(t|\mathrm{FWRR})}$). (c) FW for grain yield *t* ${\hat{y}}_{i(t|\mathrm{FW})}$). (d) FWRR for grain yield ${\tilde{y}}_{i(t|\mathrm{FWRR})}$.

In oats (Figure 1 and Figures S3–S6), training sets with a small number of environments (i.e., two or five environments) showed a higher variability in estimation using FW and prediction with FWRR, and with FRWW showing less dispersion in the prediction of ${\tilde{b}}_{i(\mathrm{FWRR})}$ with two and five environments than FW. As we increased the number of environments used to train the model, variability in the results among models decreased (Figures 1, 2, 3). In the oat datasets, changes in the mean environmental variance, as well as the variability in ${\hat{\beta }}_{i(t|\mathrm{FW})}$ and ${\tilde{b}}_{i(\mathrm{FWRR})}$, were marginal once more than 20 environments were included, with RFWW converging slightly faster toward ${\hat{\beta }}_{i(\mathrm{FW})}$ than FW.

We observed similar patterns in the barley and wheat datasets with respect to the number of environments and the variance in the means across environments (Figures 2 and 3). Increasing the number of environments led to higher predictive ability of ${\hat{\beta }}_{i(\mathrm{FW})}$ and ${\tilde{b}}_{i(\mathrm{FWRR})}$; however, gains were minimal beyond 15 environments in the barley and wheat datasets and beyond 20 environments in the oat dataset (Figures 1a, 2, and 3). For the barley and wheat datasets, when ${\hat{\beta }}_{i(t|\mathrm{FW})}$ and ${\tilde{b}}_{i(t|\mathrm{FWRR})}$ were used to predict grain yield in environments not included in the model, the average predictive ability was 0.33 (range: 0–0.62) in barley (Figure 2) and 0.52 (range: 0.05–0.82) in wheat (Figure 3).

When the number of environments per genotype is small, but many environments include related genotype, FWRR models—that borrow information from genetically related individuals—reduce the variance of genotype‐specific parameter estimates by increasing the accuracy. Using phenotypic information from relatives can therefore improve predictive accuracy, particularly when relatives share similar response patterns (Figure 1c).

### 3.2 Effect of contrasting testing environments

The variability in the predictive ability of grain yield stability decreases as more environments are used in the prediction across all three datasets (Figures 1, 2, 3). The accuracy of the stability estimates ${\hat{\beta }}_{i(\mathrm{FW})}$ and prediction ${\tilde{b}}_{i(\mathrm{FWRR})}$ increases with greater variance among environmental means, particularly when a small number of environments is evaluated. Furthermore, some subsets of two or five environments yield stability estimates (${\hat{\beta }}_{i(t|\mathrm{FW})}$ or ${\tilde{b}}_{i(t|\mathrm{FWRR})}$) within 5% of those obtained from the full dataset (${\hat{\beta }}_{i(\mathrm{FW})}$), outperforming subsets with more environments but lower variance among environmental means (Figure 1 in oat, Figure 2 in barley, and Figure 3 in wheat).

Finally, we compared the predictive ability of each location by sampling all pairs of locations and evaluating their performances. For both barley and wheat, some locations show higher predictive abilities than others (Figures S7 and S8). Those locations were usually extreme performers for grain yield (i.e., with higher or lower grain yield performance; Figure S9) including Huntley, MT, in barley, and Maule, FR, in wheat.

### 3.3 FWRR stability prediction

**FIGURE 4:**
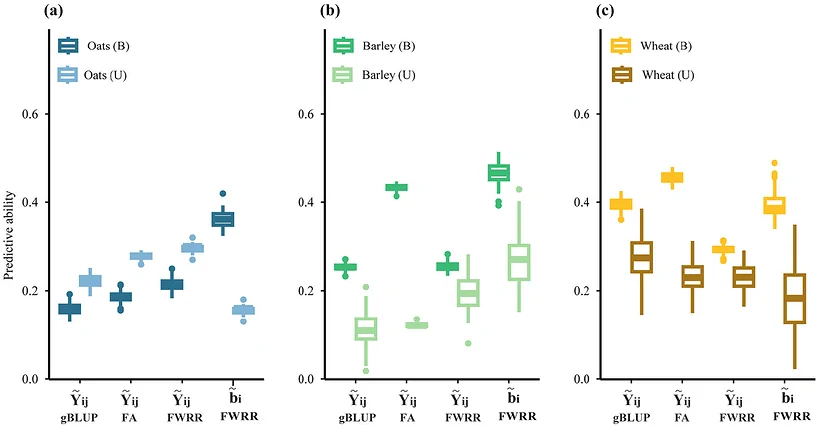
Predictive ability for grain yield using genomic best linear unbiased predictor (gBLUP) (${\tilde{y}}_{ij(\mathrm{gBLUP})}$), FA (${\tilde{y}}_{ij(\mathrm{FA})}$), and Finlay–Wilkinson random regression (FWRR) (${\tilde{y}}_{ij(\mathrm{FWRR})}$) models, and predictive ability for grain yield stability predicted using FWRR (${\tilde{b}}_{i(\mathrm{FWRR})}$). (a) Balanced oats (with >20 environments; B—dark blue), unbalanced oats (with >3 environments; U—light blue). (b) Balanced barley (B—green) and unbalanced barley (U—light green). (c) Balanced wheat (B—gold) and unbalanced wheat (U—ochre).

Moderate‐to‐high predictive ability was achieved using FWRR to predict stability ${\tilde{b}}_{i(\mathrm{FWRR})}$ across both balanced and unbalanced training datasets. Predictive abilities ranged from moderate to high in balanced datasets, with values of 0.36 (0.32–0.49) in oats, 0.47 (0.39–0.51) in barley, and 0.39 (0.34–0.49) in wheat (Figure 4). Unbalanced datasets showed lower but still meaningful predictive ability, ranging from 0.16 (0.13–0.18) in oats, 0.11 (0.02–0.21) in barley, and 0.18 (0.02–0.35) in wheat (Figure 4, Table S1). In the case of the balanced oats dataset, each genotype is still evaluated in a different number of environments, and therefore, both ${\hat{\beta }}_{i(\mathrm{FW})}$ and ${\tilde{b}}_{i(\mathrm{FWRR})}$ values have a wider range in oats than in the balanced datasets for barley and wheat (Figure 4, Table 2 and Table S1).

**TABLE 2:** Range of Finlay−Wilkinson (FW) estimated slope ${\hat{\beta }}_{i(\mathrm{FW})}$, Finlay–Wilkinson random regression (FWRR) predicted slope ${\tilde{b}}_{i(\mathrm{FWRR})}$, and 10‐fold cross‐validation (CV1) FWRR predicted slope ${\tilde{b}}_{i(\mathrm{cv}1|\mathrm{FWRR})}$ for oats, barley, and wheat datasets.

Dataset	${\hat{\beta }}_{i(\mathrm{FW})}$	${\tilde{b}}_{i(\mathrm{FWRR})}$	${\tilde{b}}_{i(\mathrm{cv}1|\mathrm{FWRR})}$
Oats balanced (>20)	0.48–1.32	0.48−1.32	0.67−1.35
Oats unbalanced (>3)	−0.6 to 2.16	−0.35 to 2.14	−0.21 to 2.37
Barley balanced	0.71−1.18	0.71−1.18	0.73−1.24
Barley unbalanced	0.37−1.35	0.38−1.34	0.51−1.54
Wheat balanced	0.57−1.22	0.57−1.22	0.80−1.13
Wheat unbalanced	0.10−1.83	0.18−1.80	−0.87 to 2.34

While the predictive ability of stability in the oat's dataset decreased from the balanced to the unbalanced dataset, the predictive ability of grain yield with both the gBLUP and the FWRR models increased (Figure 4), probably due to an increase in population size. Basically, the unbalanced oat dataset includes a higher number of genotypes than the balanced dataset. In the case of both wheat and barley, the balanced datasets outperformed the unbalanced datasets for all the parameters (Figure 4).

Finally, predictions of grain yield with the FWRR model (${\tilde{y}}_{ij(\mathrm{FWRR})}$) outperformed the grain yield predictions using the gBLUP (${\tilde{y}}_{ij(\mathrm{gBLUP})}$) and the FA (${\tilde{y}}_{ij(\mathrm{FA})}$) in both oat datasets, with the unbalanced showing the highest predictive ability (Figure 4). In barley and wheat, the FA model outperformed both the FWRR and the gBLUP in the balanced dataset, whereas the FWRR outperformed the FA model in the unbalanced dataset (Figure 4).

## 4 DISCUSSION

### 4.1 Effect of the number of environments on stability estimation

Our results suggest that once at least 20 environments are used to train the model, the estimation of ${\hat{\beta }}_{i(\mathrm{FW})}$ stabilizes and converges around the best known ${\hat{\beta }}_{i(\mathrm{FW})}$ estimator (i.e., ${\hat{\beta }}_{i}$ calculated using the full dataset, Figure 1). In the case of FWRR (Figure 1c), which leverages information from related genotypes evaluated in other environments, fewer environments are necessary to reach the same Root mean squared error (Figure 1). Increasing the number of environments and/or the degree of relatedness between training and testing populations is expected to improve the predictive ability (Neyhart et al., 2025). Therefore, FWRR opens the possibility of reducing the number of environments in which a genotype is evaluated to reliably characterize grain yield stability, while leveraging historical datasets and temporal depth.

Simulation studies on physiological traits in wheat found that at least 150 environments were needed to characterize stability accurately (Wang et al., 2023). We attribute the differences between Wang et al. (2023) and our findings to the higher diversity of the datasets simulated by Wang et al. (2023), which was higher than that of a typical breeding population. In breeding populations, there is strong selection for both yield and dynamic stability, which likely imposes a bias toward stable genotypes. This could have reduced the genetic diversity for these traits in our training datasets, reducing the number of environments required.

Similarly, in corn, estimates suggest that 50–200 trials are required to assess grain yield stability accurately (Piepho, 1998b). However, these results are based on empirical calculations over balanced trials with homogenous variance and thus are conservative in their estimation. We believe our datasets represent the sampled environmental variance.

On the other hand, Yan et al. (2015) developed a method to estimate the number of environments required to understand GEIs in a dataset of environments for a group of genotypes that depends on the trait (genetic variance, heritability), the quality of the trials (error variance, repeatability), the mega‐environment structure (environmental variance, repeatability), and the desired precision. In oats, Yan et al. (2015) found that, on average, 21 (15–34) environments are necessary to model GEI structure in grain yield when mega‐environment structure is present but not accounted for. At the same time, the number of environments required for accurate grain yield estimation is reduced to four to six environments per mega‐environment when the mega‐environment structure is modeled, due to higher heritability within mega‐environments (Yan et al., 2015). This is mainly because in linear regressions, a single extreme year can have a disproportionate impact on the results when the number of environments is small, as it can strongly influence the slope of the regression line (Westcott, 1986). Our results show that ${\hat{\beta }}_{i(\mathrm{FW})}$ and ${\tilde{b}}_{i}$ converge to the best‐known value for the slope when around 20 environments are included, which falls within the range reported by Yan et al. (2015) without modeling the mega‐environment structure. These findings indicate that, when using FW, stability estimation requires substantial accumulation of multi‐environment data. As an alternative, FWRR enables the prediction of stability for genotypes lacking phenotypic data by leveraging information from related genotypes evaluated across a broader range of environments, offering a practical strategy to accelerate selection decisions.

Our results show that both the number of environments and the variance among the environmental means affect the estimation of ${\hat{\beta }}_{i}$. When the number of environments available is limited (i.e., between two and five environments), the combination of environments that provides the maximum variance among the environmental means will result in a more accurate estimation of stability. This is expected due to the properties of linear regression, in which the standard error of the regression coefficient is inversely proportional to the variance of the predictor (Kutner, 2005). Being able to exploit contrasting environmental means in breeding programs can optimize resource allocation through early stability testing using a few highly informative environments and targeting locations based on those breeding objective (Neyhart et al., 2022); for example, using one environment with high yield and one with moderately low yield, a large set of genotypes can be screened in the early stages. A breeding program could also design sparse testing schemes that pair environments with high variance in their means. In our analysis, several combinations of environments could accurately estimate stability, leaving the breeder room to design the testing scheme based on their knowledge of the TPE and the feasibility of the testing. Our results suggest that selecting environments that maximize the variance among their means is the best strategy to extract the most information on stability when breeders do not have access to genomic selection. As information accumulates and more environments are added, the marginal improvement from individual environments decreases, and the overall quality of the stability estimation improves.

### 4.2 FWRR and stability prediction

Our results suggest that predictions from FWRR do not differ from those from FW when using the full dataset and more than 20 environments in unbalanced datasets. We hypothesize that the minimal differences between methods under these conditions arise because ${\hat{\beta }}_{i}$ is already robustly estimated; therefore, incorporating additional information from related genotypes provides little new information, similar to the diminishing returns of adding extra environments. In contrast, when fewer than 20 environments are available, FWRR has a clear advantage, as it capitalizes on information from related genotypes evaluated in different environments, effectively increasing the amount of data available to estimate ${\tilde{b}}_{i}$. Moreover, FWRR enables the prediction of grain yield stability for genotypes that have not been phenotyped yet by leveraging phenotypic information from related individuals.

Genomic prediction creates an opportunity to implement genomic‐informed selection for stability early in a breeding program, during population development, and before extensive multi‐environment testing. Early selection for stability would allow breeders to prioritize genotypes with a higher likelihood of stable performance before the first grain yield evaluations, thereby focusing resources on the most promising lines. Ultimately, this approach can improve selection efficiency, optimize resource allocation, and reduce the time required to release stable cultivars.

The predictive ability for values in oats genotypes tested in over 20 environments was 0.36 (0.32–0.42), which is within the range of reported values for grain yield in oats (Sandro et al., 2024). The predictive ability for ${\tilde{b}}_{i(\mathrm{cv}1|\mathrm{FWRR})}$ in an unbalanced dataset of oats was only 0.16 (0.14–0.19). This pattern is consistent with the observed variability in the predictive ability of ${\tilde{b}}_{i(\mathrm{FWRR})}$ prediction when fewer than 10 environments were used to predict ${\tilde{b}}_{i(\mathrm{FWRR})}$ in balanced datasets (Figures 2 and 3). Our inability to accurately estimate the true value of ${\hat{\beta }}_{i(\mathrm{FW})}$ could be explained by the strong weight of the per se estimates of the slope used, compared to the weights of the slope of related genotypes. If the genotypes are well connected to the training population, a prediction based on their relatives may be more trustworthy than an estimate from a few biased environments. Additionally, when comparing ${\tilde{b}}_{i(\mathrm{FWRR})}$ against a ${\hat{\beta }}_{i(\mathrm{FW})}$ estimated with fewer than 20 environments, we are relying on the use ${\hat{\beta }}_{i(\mathrm{FW})}$, which showed high variability and represents a less reliable “true” value. Consequently, the resulting predictive abilities could be biased and underestimated, which means that more data are needed to make a fair comparison between ${\tilde{b}}_{i(\mathrm{FWRR})}$ and ${\hat{\beta }}_{i(\mathrm{FW})}$ when the number of environments is limited. In predicting ${\tilde{b}}_{i(\mathrm{FWRR})}$, information from relatives is leveraged, effectively drawing information from a larger number of environments. In consequence, prediction is weighted by the available data from relatives, making ${\tilde{b}}_{i(\mathrm{FWRR})}$ more stable and accurate than estimates of ${\hat{\beta }}_{i(\mathrm{FW})}$, which are limited to the data observed for each genotype. However, this improvement did not translate into higher predictive ability in our study, since the comparison was made between potentially high‐quality ${\tilde{b}}_{i(\mathrm{FWRR})}$ prediction and a low‐quality ${\hat{\beta }}_{i(\mathrm{FW})}$ estimation. Grain yield prediction benefits from higher accuracy. ${\tilde{b}}_{i(\mathrm{FWRR})}$ Moreover, the observed grain yield values used for validation are relatively accurate, even with fewer trials. Therefore, we may be comparing the predictive abilities for grain yield to accurate “truth” values but the predictive ability for stability to inaccurate “truth” values. The improvement in grain yield prediction is also explained by the increasing number of related genotypes in the dataset, resulting in higher predictive ability (Heslot et al., 2015). In FWRR, increasing the number of genotypes similarly increases the number of relatives in the dataset, improving the estimation of genotypic values for each environment. Nevertheless, this improvement does not translate into better slope predictions, due to the issues explained earlier.

For our balanced datasets, the value of predictive ability for ${\tilde{b}}_{i(\mathrm{cv}1|\mathrm{FWRR})}$ in barley was 0.47 (0.39−0.51), which is within the range previously reported by Neyhart et al. (2025). In wheat, the value of predictive ability for ${\tilde{b}}_{i(\mathrm{cv}1|\mathrm{FWRR})}$ was 0.39 (0.33–0.49), which is also within the range previously reported by Olivoto et al. (2019). Even with relatively small datasets, it is possible to predict grain yield stability with moderate predictive ability in cereals, both in balanced and unbalanced datasets.

### 4.3 FWRR and grain yield prediction

The predictive abilities obtained using ${\tilde{b}}_{i(\mathrm{cv}1|\mathrm{FWRR})}$ to model grain yield in unobserved genotypes are similar to the predictive abilities reported in the literature when environmental effects are included in the model (Lado et al., 2016; Rebollo et al., 2023). The oat and barley datasets have approximately 10% higher environmental variance than the wheat dataset, which could help to explain the inferior relative performance of the FW models in wheat. A higher proportion of similar environments implies a smaller variance of *x* used for the FWRR slope prediction, increasing the standard error of the predictions. We believe that the improvement in predictive ability for ${\tilde{y}}_{ij}$ when comparing the oat balanced and unbalanced training datasets is due to the increase in the number of genotypes that are closely related, which increase the amount of information and connectivity of the training dataset, improving the precision of predictions (Berro et al., 2019; Heslot et al., 2015). The moderate predictability of grain yield stability is an important result because it allows us to apply genomic prediction for grain yield stability early in the process of population development, long before we have phenotypic information from multi‐environment trials for the genotype.

We hypothesize that the differences in the patterns observed for the prediction of ${\tilde{y}}_{ij(\mathrm{FWRR})}$, ${\tilde{y}}_{ij(\mathrm{gBLUP})}$, and ${\tilde{y}}_{ij(\mathrm{FA})}$ were mainly due to the diversity of the environments in each training dataset. For wheat, the environments in the training dataset are more similar and exhibit a high level of correlation (Figure S1; Rincent et al., 2019). In our oat and barley datasets, we observed a more complex environmental structure, with a larger proportion of the variance associated with environmental variance than with GEIs (Figure S10; Neyhart et al., 2022; Sandro et al., 2024).

## 5 CONCLUSIONS

In conclusion, when there is balanced phenotypic information available, FW and FWRR do not differ in their values of ${\hat{\beta }}_{i(FW)}$ and ${\tilde{b}}_{i(FWRR)}$. The results suggest that adding more environments improves stability estimation until about 20 environments are included in the training data, after which the accuracy of the estimation plateaus. When fewer than 20 environments are available, the contrast among environmental means is the most important factor affecting accuracy in stability estimates. The stability of new genotypes without grain yield information can be accurately predicted using FWRR if related genotypes are included in the dataset and tested in at least 20 environments. Overall, we demonstrated that historical datasets are useful for informing prediction breeding and for designing more efficient testing strategies to select complex traits such as grain yield stability.

## AUTHOR CONTRIBUTIONS

**Pablo Sandro**: Data curation; formal analysis; investigation; software; visualization; writing—original draft; writing—review and editing. **Justin Blancon**: Data curation; formal analysis; methodology; supervision; writing—original draft; writing—review and editing. **Jeffrey Neyhart**: Data curation; writing—review and editing. **Julie Dawson**: Methodology; writing—review and editing. **Lucia Gutiérrez**: Conceptualization; data curation; formal analysis; funding acquisition; investigation; methodology; project administration; resources; supervision; visualization; writing—original draft; writing—review and editing.

## CONFLICT OF INTEREST STATEMENT

The authors declare no conflicts of interest.

## Supporting information




**Supplementary Materials**.

## Data Availability

All oat data are available in the T3/Oat database (available at https://oat.triticeaetoolbox.org). Barley data are available on the T3 database (https://barley.triticeaetoolbox.org) under the “UMN Spring 2‐row MET” experiment name. Wheat data are not publicly available.
