# Supplementary material for: Finlay–Wilkinson random regression for yield and yield stability prediction in cereals
Source: Plant Genome. 2026 Sep 9;19(3):e70269. doi: 10.1002/tpg2.70269 (PMC13555820; doi:10.1002/tpg2.70269)
Supplement: Supplementary file 1 [file TPG2-19-e70269-s001.docx]

Supplementary material

# Finlay-Wilkinson random regression for yield and yield stability prediction in cereals

Pablo Sandro, Justin Blancon, Jeffrey Neyhart, Julie Dawson, Lucia Gutiérrez

Affiliations:

Pablo Sandro - Department of Plant and Agroecosystem Sciences, University of Wisconsin- Madison, WI 53706, USA.

Justin Blancon - UMR GDEC, INRAE, Université Clermont Auvergne, Clermont-Ferrand, France

Jeffrey Neyhart - USDA, Agricultural Research Service, Genetic Improvement for Fruits & Vegetables Laboratory, Chatsworth, NJ 08019, USA.

Julie Dawson - Department of Plant and Agroecosystem Sciences, University of Wisconsin- Madison, WI 53706, USA - [dawson@hort.wisc.edu](mailto:dawson@hort.wisc.edu)

Lucia Gutiérrez - Department of Plant Breeding, Swedish University of Agricultural Sciences, Alnarp, Sweden - [lucia.gutierrez.chacon@slu.se](mailto:lucia.gutierrez.chacon@slu.se)

Corresponding author: Lucia.Gutierrez.Chacon@slu.se

**Supplementary Figure 1**. Environments distribution per training population **a.** Oats (each blue dots represent a location), Midwest of the United States (Sandro et al. 2024). **b.** Barley (each green dot represents a location), Midwest of the United States and south of Canada (Neyhart et al. 2019). **c.** Wheat (each golden dot represents a location), France west and central regions (Rincet et al. 2019).

**Supplementary Figure 2.** Heritability for barley, oats, and wheat trials.

**Supplementary Figure 3.** Cultivar Ogle’s $\hat{\beta}_{i(t|FW)}$ variation on the number of environments, variance among environment means. The red lines represent the value of $\hat{\beta}_{i(t|FW)}$ estimated using all environments available for the line (480 environments). **a.** Coefficient of variation for $\hat{\beta}_{i(t|FW)}$ per number of environments used to fit the model. **b.** $\hat{\beta}_{i(t|FW)}$ against the number of environments used to fit the model. **c.** $\hat{\beta}_{i(t|FW)}$ versus variance among means of environments for 100 training populations formed by 2, 5, 20, 30, 50, 100, and 200 environments.

**Supplementary Figure 4**. Deon’s $\hat{\beta}_{i(t|FW)}$ variation on the number of environments, variance among means of environments used to estimate $\hat{\beta}_{i(t|FW)}$. The red lines represent the value of $\hat{\beta}_{i(t|FW)}$(1.05) estimated using all environments available for the line (235 environments). **a.** Coefficient of variation for $\hat{\beta}_{i(t|FW)}$ per number of environments used to fit the model. **b.** $\hat{\beta}_{i(t|FW)}$ against the number of environments used to fit the model. **c.** $\hat{\beta}_{i(t|FW)}$ variance among the means for 100 iterations of 2, 5, 20, 30, 50, 100, and 200 environments used to fit the model.

**Supplementary Figure 5.** Horsepower’s $\hat{\beta}_{i(t|FW)}$variation on the number of environments, variance among means of environments used to fit the model. The red lines represent the value of $\hat{\beta}_{i(t|FW)}$ (1.00) estimated using all environments available for the line (232 environments). **a.** Coefficient of variation for $\hat{\beta}_{i(t|FW)}$ per number of environments used to fit the model. **b.** $\hat{\beta}_{i(t|FW)}$against the number of environments used to fit the model. **c.** $\hat{\beta}_{i(t|FW)}$variance of the means of environments for 100 sets of 2, 5, 20, 30, 50, 100, and 200 environments used to fit the model.

**Supplementary Figure 6.** Clintland64’s $\hat{\beta}_{i(t|FW)}$ variation in the number of environments, variance of the means of environments used to fit the model. The red lines represent the value of $\hat{\beta}_{i(t|FW)}$ (0.87) estimated using all environments available for the line (265 environments). **a.** Coefficient of variation for $\hat{\beta}_{i(t|FW)}$ per number of environments used to fit the model. **b.** $\hat{\beta}_{i(t|FW)}$ against the number of environments used to fit the model. **c.** $\hat{\beta}_{i(t|FW)}$ versus variance of mean of environments for 100 sets of 2, 5, 20, 30, 50, 100, and 200 environments used to fit the model

**Supplementary Figure 7.** Barley predictive ability by location for all possible pairs of environments in which each location participated. Some locations consistently give predictions over the mean, suggesting that these locations should be used in early testing (Locations abbreviation code: BCW - Buffalo county, WI; HNY – Ithaca site 1, NY; HNY – Ithaca site 2, NY; AWI – Arlington, WI; CHM – Chatham, MI; COH – Wooster, OH; FND – Fargo, ND; MWI – Madison, WI; EON – Elora, ON; BZD – Bozeman site 1, MT; CRM – Crookston, MN; STP - St_Paul, MN; PQC – Princeville, QC; BZI – Bozeman site 2, MT; WLI – West Lafayette, IN; AID – Aberdeen, ID; HTM – Huntley, MT).

**Supplementary Figure 8.** Wheat predictive ability by location for all possible pairs of environments in which the location participated. The red dotted line represents the mean predictive ability for all location combinations. Some locations consistently give predictions over the mean performance; this means that they are better than the other locations (locations abbreviation code: MOMcoi – Coings, DEScap – Cappelle, - INRcle - Clermont-Ferrand, BAYvra – Vraux, ARVcha – Champigny, RNlou – Louville, INRmon – Mons, RNcha – Chalons, CAUrec, - Réclainville, SYNand – Andelu, MOMall – Allonnes, ARVgre, - Gréoux, LIMver – Verneuil, SECmau – Maule)

**Supplementary Figure 9**. Standardized grain yield by standardized environment means for oats (a.), barley (c.) and wheat (e.) datasets, and standardized environments means distributions for oats (b.), Barley (d.), and wheat (f.) datasets.

**Supplementary Figure 10**. Variance decomposition for oats, barley and wheat training population.

**Supplementary Table 1.** Predictive ability for gBLUP predicted grain yield $\tilde{y}_{ij(cv1|gBLUP)}$ calculated as cor($\hat{y}_{ij}$, $\tilde{y}_{ij(cv1|gBLUP)}$), RFW predicted grain yield $\tilde{y}_{ij(cv1|RFW)}$ calculated as cor($\hat{y}_{ij}$, $\tilde{y}_{ij(cv1|RFW)}$), and RFW slope prediction $\tilde{b}_{i(cv1|RFW)}$ calculated as cor($\hat{\beta}_{i(FW)}$, $\tilde{b}_{i(cv1|RFW)}$). Range is shown in parentheses.

| **Dataset** | $\boldsymbol{cor(}{\hat{\boldsymbol{y}}}_{\mathbf{ij}}\boldsymbol{,}{\tilde{\boldsymbol{y}}}_{\mathbf{i(}\boldsymbol{cv}\mathbf{1\vert GBLUP)}}\boldsymbol{)}$ | $\boldsymbol{cor(}{\hat{\boldsymbol{y}}}_{\mathbf{ij}}\boldsymbol{,}{\tilde{\boldsymbol{y}}}_{\mathbf{i(}\boldsymbol{cv}\mathbf{1\vert RFW)}}\boldsymbol{)}$ | $\boldsymbol{cor(}{\hat{\boldsymbol{\beta}}}_{\mathbf{i(}\boldsymbol{FW}\mathbf{)}}\boldsymbol{,}{\tilde{\boldsymbol{b}}}_{\mathbf{i(}\boldsymbol{cv}\boldsymbol{1}\mathbf{\vert RFW)}}\boldsymbol{)}$ |
| --- | --- | --- | --- |
| Oats unbalanced (>20) | 0.16 (0.13, 0.20) | 0.21 (0.18, 0.25) | 0.36 (0.32, 0.42) |
| Oats unbalanced (>3) | 0.22 (0.19, 0.26) | 0.30 (0.28, 0.32) | 0.16 (0.13, 0.18) |
| Barley balanced | 0.25 (0.23, 0.27) | 0.25 (0.23, 0.28) | 0.47 (0.39, 0.51) |
| Barley unbalanced | 0.11 (0.02, 0.21) | 0.20 (0.08, 0.28) | 0.27 (015, 0.43) |
| Wheat balanced | 0.40 (0.36, 0.43) | 0.29 (0.27, 0.31) | 0.39 (0.34, 0.49) |
| Wheat unbalanced | 0.28 (0.15, 0.39) | 0.23 (0.16, 0.29) | 0.18 (0.02, 0.35) |

**Core ideas**

1. Finlay-Wilkinson regression estimates stability with high accuracy when fit with 20 or more environments.

2. Finlay-Wilkinson random regression predicts stability with high accuracy for genotypes with no phenotypic data.

3. Pairs with high variance among environment means are as accurate in stability estimation as 20 environments.
